# Analysis of Cancer Mutation Signatures in Blood by a Novel Ultra-Sensitive Assay: Monitoring of Therapy or Recurrence in Non-Metastatic Breast Cancer

**DOI:** 10.1371/journal.pone.0007220

**Published:** 2009-09-28

**Authors:** Zhenbin Chen, Jinong Feng, Carolyn H. Buzin, Qiang Liu, Lawrence Weiss, Kemp Kernstine, George Somlo, Steve S. Sommer

**Affiliations:** 1 Department of Molecular Genetics, City of Hope National Medical Center, Duarte, California, United States of America; 2 Department of Molecular Diagnosis, City of Hope National Medical Center, Duarte, California, United States of America; 3 Department of Anatomic Pathology, City of Hope National Medical Center, Duarte, California, United States of America; 4 Division of Surgery, City of Hope National Medical Center, Duarte, California, United States of America; 5 Department of Medical Oncology, City of Hope National Medical Center, Duarte, California, United States of America; 6 MEDomics, LLC, Azusa, California, United States of America; Brunel University, United Kingdom

## Abstract

**Background:**

Tumor DNA has been shown to be present both in circulating tumor cells in blood and as fragments in the plasma of metastatic cancer patients. The identification of ultra-rare tumor-specific mutations in blood would be the ultimate marker to measure efficacy of cancer therapy and/ or early recurrence. Herein we present a method for detecting microinsertions/deletions/indels (MIDIs) at ultra-high analytical selectivity. MIDIs comprise about 15% of mutations.

**Methods and Findings:**

We describe MIDI-Activated Pyrophosphorolysis (MAP), a method of ultra-high analytical selectivity for detecting MIDIs. The high analytical selectivity of MAP is putatively due to serial coupling of two rare events: heteroduplex slippage and mis-pyrophosphorolysis. MAP generally has an analytical selectivity of one mutant molecule per >1 billion wild type molecules and an analytical sensitivity of one mutant molecule per reaction. The analytical selectivity of MAP is about 100,000-fold better than that of our previously described method of Pyrophosphorolysis Activated Polymerization-Allele specific amplification (PAP-A) for detecting MIDIs. The utility of this method is illustrated in two ways. 1) We demonstrate that two *EGFR* deletions commonly found in lung cancers are not present in tissue from four normal human lungs (10^7^ copies of gDNA each) or in blood samples from 10 healthy individuals (10^7^ copies of gDNA each). This is inconsistent, at least at an analytical sensitivity of 10^−7^, with the hypotheses of (a) hypermutation or (b) strong selection of these growth factor-mutated cells during normal lung development leads to accumulation of pre-neoplastic cells with these *EGFR* mutations, which sometimes can lead to lung cancer in late adulthood. Moreover, MAP was used for large scale, high throughput “gene pool” analysis. No germline or early embryonic somatic mosaic mutation was detected (at a frequency of >0.3%) for the 15/18 bp *EGFR* deletion mutations in 6,400 individuals, suggesting that early embryonic *EGFR* somatic mutation is very rare, inconsistent with hypermutation or strong selection of these deletions in the embryo. 2) The second illustration of MAP utility is in personalized monitoring of therapy and early recurrence in cancer. Tumor-specific *p53* mutations identified at diagnosis in the plasma of six patients with stage II and III breast cancer were undetectable after therapy in four women, consistent with clinical remission, and continued to be detected after treatment in two others, reflecting tumor progression.

**Conclusions:**

MAP has an analytical selectivity of one part per billion for detection of MIDIs and an analytical sensitivity of one molecule. MAP provides a general tool for monitoring ultra-rare mutations in tissues and blood. As an example, we show that the personalized cancer signature in six out of six patients with non-metastatic breast cancer can be detected and that levels over time are correlated with the clinical course of disease.

## Introduction

The ability to detect exceedingly rare somatic mutations associated with cancers will help elucidate mechanisms of carcinogenesis and monitor early recurrent cancer in personalized medicine. Spontaneous mutation frequency is as low as 1×10^−8^ and 2.1×10^−6^ in human normal and cancerous tissues, respectively [1].

The analytical selectivity (see Terminology in **Methods**) of conventional sequencing or massively parallel DNA pyrosequencing is currently one part in ten or one part in 100, respectively [2]. Previous analytic methods generally have analytical selectivities of 10^2^–10^3^, with the exception of BEAMing and MutEx/ACB-PCR for a limited subset of restriction sites [3], [4]. Recently, Pao and Ladanyi [5] compared 13 methods for detecting the common 15 bp epidermal growth factor receptor (*EGFR*) deletion in lung cancers (including Loop-hybrid mobility shift assay, Cycleave PCR, PCR-RFLP and length analysis, MALDI-TOF MS–based genotyping, PNA-LNA PCR clamp, Scorptions ARMS, Mutant-enriched PCR). Of these, the most sensitive method, SMart Amplification Process, SMAP [6], had an analytical selectivity of 1 in 10^3^.

Pyrophosphorolysis-Activated Polymerization -Allele specific amplification (PAP-A) is a sensitive and selective method for DNA amplification to detect ultra rare mutations [7]. The method utilizes allele-specific oligonucleotides that are blocked at the 3′ end by a dideoxy nucleotide. These “sleeping beauties” are inert until activated on their cognate template by the “kiss” of pyrophosphorolysis, allowing extension to occur. PAP-A has a potential analytical selectivity of 3.3×10^11^ because false positives can occur only if two independent rare events occur in series: mismatch pyrophosphorolysis and misincorporation (mis-polymerization) at the first polymerized nucleotide (Fig. S1A). PAP-A has an actual analytical selectivity of 10^4^∼10^5^ because of polymerase misincorporation within the extension product from the opposite primer (bypass reaction) [7].

Bi-directional PAP-A (Bi-PAP-A) was developed to eliminate the bypass reaction, and thereby increase analytical selectivity, by using two blocked primers that overlap at one base [8], [9] (Fig. S1B). It has an analytical selectivity of >1×10^7^ for certain single-base substitutions (G>C, C>G, A>T, or T>G). However, the high baseline frequency of the deaminated cytosine and 8-oxo-guanidine in genomic DNA limits the analytical selectivity of C>T or G>T by Bi-PAP-A assays to >10^4^ and >10^5^, respectively.

When PAP-A primers are designed to detect microinsertions/deletions/indels (MIDIs), the observed analytical selectivity is less than one part in 10^5^ for reasons that are unclear (Fig. S1C). Therefore, we developed MIDI-Activated Pyrophosphorolysis (MAP), a method with a MIDI analytical selectivity that is generally 100,000 fold greater (≥1×10^9^) than in PAP-A. MAP is a seemingly simple modification of PAP-A in which the blocked oligonucleotides (“sleeping beauties”) have multiple mismatches to the wild type sequence. In MAP, false positives arise by the serial coupling of a heteroduplex slippage event followed by pyrophosphorolysis of mismatched heteroduplexes. Unlike PAP-A, there is no requirement for misincorporation.

We illustrate the utility of MAP for addressing biological questions by: i) testing the hypothesis that the common 15 and 18 bp microdeletions in the *EGFR* gene in non-small cell lung cancers derive from pre-neoplastic mutations selected during lung development [10] and ii) monitoring of therapy and early recurrence by detecting personalized cancer mutation signatures in the blood of women with stage II and III breast cancers. Herein, we demonstrate that analysis of plasma can reliably detect cancer mutation signatures in six women with stage II and III breast cancer.

## Methods

### Terminology

#### MIDI

Microinsertion, deletion or indel; an insertion, deletion or indel that results in a gain or loss of 1 to 50 nucleotides [11].

#### Pyrophosphorolysis

The removal of the 3′ terminal nucleotide by DNA polymerase in the presence of pyrophosphate (PPi) to generate the nucleotide triphosphate. Pyrophosphorolysis is the reverse of DNA polymerization.

#### MAP

MIDI- activated pyrophosphorolysis.

#### PAP

Pyrophosphorolysis-activated polymerization. Variants of PAP include allele-specific PAP (PAP-A) and bi-directional PAP (bi-PAP-A) [7].

#### Sleeping Beauties (P*)

An oligonucleotide with a blocked 3′ terminus that is not directly extendable but is activable by pyrophosphorolysis [7] (see Fig.S1A).

#### Analytical Sensitivity

The minimum copy number of a template that generates a detectable product when P* matches the mutant template. It is determined by serial dilution of the mutant DNA molecules.

#### Analytical Specificity

The maximum copy number of the mismatched template that does not result in a detectable product when P* mismatches the wild-type template. It is determined by serial dilution of the wild-type molecules.

#### Analytical Selectivity

The ratio of analytical specificity to analytical sensitivity.

#### Indel

A mutation resulting in a co-localized insertion and deletion with a net gain or loss of nucleotides.

#### Tandem-base mutation (TBM)

A mutation that results in base changes at adjacent nucleotides [12], [13].

#### Doublet

A mutant containing two nonadjacent mutations [14].

### Primer design and synthesis of P*

Standard primers were designed to amplify wild type or mutant segments with Oligo 5 software (National Biosciences) (Table S1).

#### MAP primers

**Figure 1:**
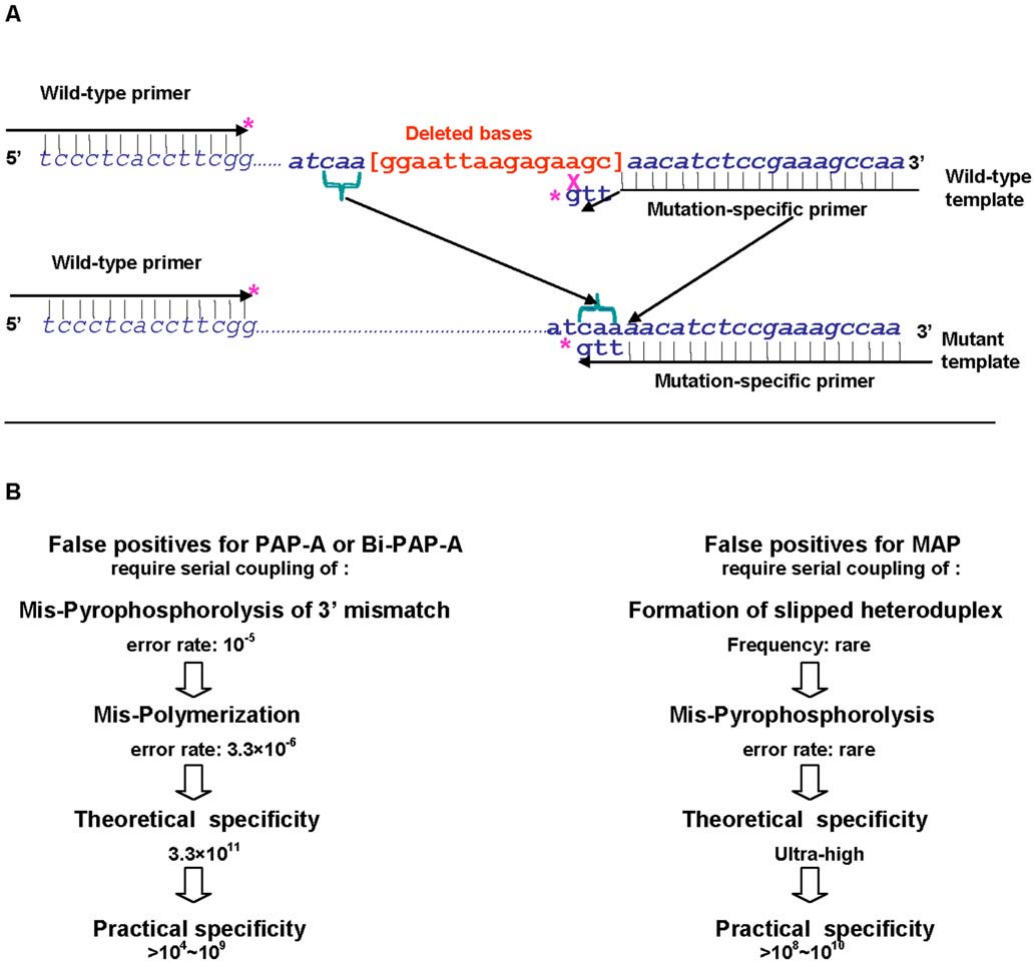
A: MAP: Introducing multiple oligonucleotide mismatch into the 3′ end of a mutation-specific blocked primer. An example of a deletion mutant sequence (the common *EGFR* 15 bp deletion) is shown below the wild-type sequence (deleted sequence in brackets, red letters). The last three bases (3′) of a mutation-specific 3′ blocked primer (upstream) are complementary to the three bases (caa) just before the 5′ end of the deletion; the primer mismatches the wild-type sequence at the three bases (agc) at the 3′ end of the deleted region. Asterisks indicate the 3′ dideoxynucleotide of the blocked primers. The “X” represents mismatch between the mutant-specific primer and wild-type sequence. B: Serial coupling of two errors underlies the ultra-high analytical selectivity of PAP and MAP. PAP-A or Bi-PAP-A and MAP derive their high analytical selectivity from serial coupling of two events, but the events differ. The practical analytical specificity for PAP-A and Bi-PAP-A is limited by side reactions such as misincorporation from the extended generic PAP primer or the presence of DNA damage products such as deaminated cytosine or 8-oxo guanidine. In contrast, false positives in MAP require the serial coupling of DNA slippage and mis-pyrophosphorolysis within this distorted DNA structure.

A pair of primers with similar Tm values, each about 30 bases in length and separated by a 50∼300 bp sequence segment, was designed for each MAP assay to detect rare deletions. Each P* primer was modified by adding a dideoxynucleotide at the 3′ terminus as described previously [15]. The mutation-specific primer mismatched the wild type sequence at two to six bases, but matched the mutant sequence at these positions (Fig. 1A). For detecting a mutation in plasma, the size of the amplicon should be <100 bp because plasma DNA is highly degraded.

#### Primers for site-directed mutagenesis

To generate mutant templates to use as positive controls, a mutation-specific primer is composed of 45-mers of mutant sequence with 10–15 nucleotides complementary to wild-type sequence at the 3′ termini for annealing to the wild-type template [16].

### Preparation of templates for MAP for testing analytical specificity and analytical sensitivity

Normal genomic DNA was isolated from blood in healthy relatives of patients with hemophilia by Puregene Genomic DNA Purification Kit (Gentra Systems, USA) and corresponding wild type segments of *EGFR* (NM_005228.3) and *EGFR2* (NM_001005862.1) were amplified. Mutant genomic DNAs were isolated from lung tumors that had been sectioned and microdissected as described previously[17]. Standard extraction protocols were optimized for low levels of DNA. Carrier nucleic acids are utilized to avoid losses due to absorption.

Four DNAs with different *EGFR* mutations [two with the common deletions (c.2235_2249del15 and c.2240_2257del18), one with a tandem base mutation (TBM; see *Terminology* in Methods) and one with a doublet] were amplified with standard primers. The artificial 14 bp deletion in exon3 in the human *EGFR* gene was generated by site-directed mutagenesis PCR to use in “control” assays. The assays for the rat *EGFR* (NM_031507.1) 15/18 bp deletions and *EGFR2* (NM_017003.2) 15 bp deletion were designed based on the homologous region in human *EGFR* (Table S1).

The above PCR products were cloned with TOPO TA Cloning kit (Invitrogen Life Technologies). Plasmid DNA with the inserted amplicon was confirmed by sequencing in both directions. Plasmids containing the wild-type sequence served as the template for the analytical specificity test. Plasmids containing mutant sequence served as the template for the analytical sensitivity test. The other positive templates with a length of 100 bp were synthesized by Sigma Company. Wild type and mutant DNAs were cloned sequentially and separately to avoid cross contamination. An analytical sensitivity assay was performed by adding a series of dilutions of 100, 10, 4, 2, 1, 0.5, 0.25 copies of subcloned mutant DNA (human or rat) or synthesized oligonucleotides. Analytical specificity was determined by adding a series of dilutions of 10^10^, 10^9^, 10^8^, 10^7^, 10^6^, 10^5^, 10^4^ copies of correspondingly subcloned wild-type DNA (human or rat).

### MAP reaction

The MAP reaction mixtures consist of 50 mM Tris-HCl (pH: 7.8, 25°C), 16 mM (NH_4_)_2_SO4, 1mM DTT, 1.5 mM MgCl_2_, 90 µM PPi, 100 µM P*, 4%DMSO, 25 µM dNTP, 4 U KlenTaq S in a total volume of 50 µl in addition to 100 ng mouse gDNA carrier. The cycling conditions were 94°C for 20 seconds, 60°C for 30 seconds, 64°C for 30 seconds, 68°C for 30 seconds, 72°C for 30 seconds, for a total of 50–55 cycles. In addition, 94°C for 2 minutes was used for the initial denaturation and 72°C for 7 minutes for the last extension. Reaction products (5 µl) were electrophoresesed through a standard 2% or 4% agarose gel with ethidium bromide. The gel was photographed under UV light by a CCD camera. The products were also submitted for sequencing for confirmation of the mutation (data not shown). Hot-start MAP was performed with Mag Hotbead (KK Biomed Corporation, Salt Lake City, Utah) to elevate the analytical selectivity of the reaction.

### Control for contamination and inhibition

In order to avoid PCR contamination [18], [19], reagents were divided into aliquots and reactions were set up in a SterilGard II hood. Seven parallel negative controls without a DNA template were assayed to rule out the possibility of contamination of the highly sensitive MAP assays. Mushroom DNA was extracted and then amplified simultaneously to test for contamination during DNA extraction.

To exclude the possibility of an inhibitor in the tested DNA, two positive controls containing the same amount of tested DNA were spiked with 10 or 4 copies of the mutant, and were amplified simultaneously to confirm that one mutant molecule can be amplified in the presence of 1.7–3.3 µg DNA (0.5–1×10^6^ copies of genome). The QIAamp DNA mini-kit was chosen due to its ability to remove inhibitors.

### Quantitative MAP based on Poisson distribution

Multiple parallel reactions (10–20) are performed per sample. Some of the parallel reactions may be negative because of no mutant template. Every positive reaction is regarded as being derived from one or more copies of mutant templates. Based on Poisson distribution, the expected average number of mutant templates per reaction is estimated using a formula (Poisson distribution) f(0) = e^−x^, where x is the average number of mutants per reaction [7]. The mutation frequency is calculated as the number of mutants (the average number of mutants × the total number of reactions) divided by the approximate total number of copies of genomic DNA contained in the 10–20 reactions per sample or per ml plasma.

### Reconstruction experiments

The human *EGFR* 15 bp deletion served as a model to investigate the relationship between the number of mismatched nucleotides and MAP analytical selectivity. To determine analytical sensitivity and analytical specificity, reconstruction experiments were conducted with 3.3 µg mouse genomic DNA (1×10^6^ copies of genomic molecules with or without spiked mutant DNA). We previously demonstrated that analytical sensitivities were similar in reconstruction experiments in the presence of 3.3 µg of human genomic DNA from cells containing the mutation of interest [9].

### Detection of EGFR deletions in normal human lung by MAP

Anonymized human lung tissues were obtained utilizing City of Hope IRB protocol 01200 for discard samples to be used in methods development and research. All four of the autopsy lung samples were obtained from patients with leukemia. DNA from blood samples from ten healthy relatives of patients with hemophilia, previously used for a different study, was also tested under the approved IRB 01200 discard sample protocol. The *EGFR* 15 and 18 bp deletions in exon19, a 14 bp artificial deletion in exon 3, and an *EGFR2* 15 bp homologous deletion were analyzed in the normal lung tissue and blood by MAP. Twelve or twenty-two parallel amplifications, containing a combined total of 10^7^ molecules of genomic DNA, were conducted simultaneously with analytical sensitivity assays, positive controls, and multiple negative controls.

### Detection of EGFR deletions in normal rat lung by MAP

For rat tissues (Fisher), the 15 and 18 bp deletions in the rat *EGFR* gene homologous to the human *EGFR* gene were analyzed in normal lung and liver tissues from 5 adults. The homologous 15 bp deletion in *EGFR2* exon 20 was used as a control in rat lung and liver tissues, as well as an artificial 15 bp deletion in *EGFR2* exon19. The primers used are shown in Table S1.

### Somatic mosaicism of the EGFR 15/18 bp deletions tested by “gene pool” analysis

DNA samples from leukocytes of 400 healthy individuals were pooled together as a group with an aggregated concentration of 200 ng/µl. MAP was utilized to detect the *EGFR* gene 15/18 bp deletions in 16 such groups, for a total of 6,400 individuals. Four µl of DNA per group was used in MAP assays; e.g., mosaics at a frequency of 1 in 300 cells were tested (2 ng DNA/individual).

### Monitoring of early recurrence of breast cancer by cancer signature mutation

Blood samples (15 ml) were collected from six patients with breast cancer and tested at intervals over a number of time points, including before therapy, before and after three cycles of adjuvant chemotherapy, just prior to surgery, and at 3, 6, 9, and 12 months' follow-up after surgery. Each patient was tested at 3 to 9 different time points over this period and followed in total for an average of about 11 months (range 8–17 months). Each patient is a participant in a City of Hope Cancer Center IRB-approved clinical trial, protocol 05015; all patients signed an informed consent for the use of their samples.

Plasma was separated from fresh or previously frozen blood by centrifugation. The amount of plasma obtained from frozen blood after removal of blood cells is equivalent to half the amount obtained from fresh blood. DNA from 1–3 ml plasma was extracted by QIAamp DNA Micro kit (Qiagen Inc.) with an addition of carrier RNA. DNA from blood cells was extracted by QIAamp Blood DNA Maxi Kit (Qiagen Inc.). The mutation test was performed on 1×10^7^ copies of genomic DNA from blood cells (about 1 ml blood).

## Results

### MAP increases analytical selectivity 100,000-fold

**Figure 2:**
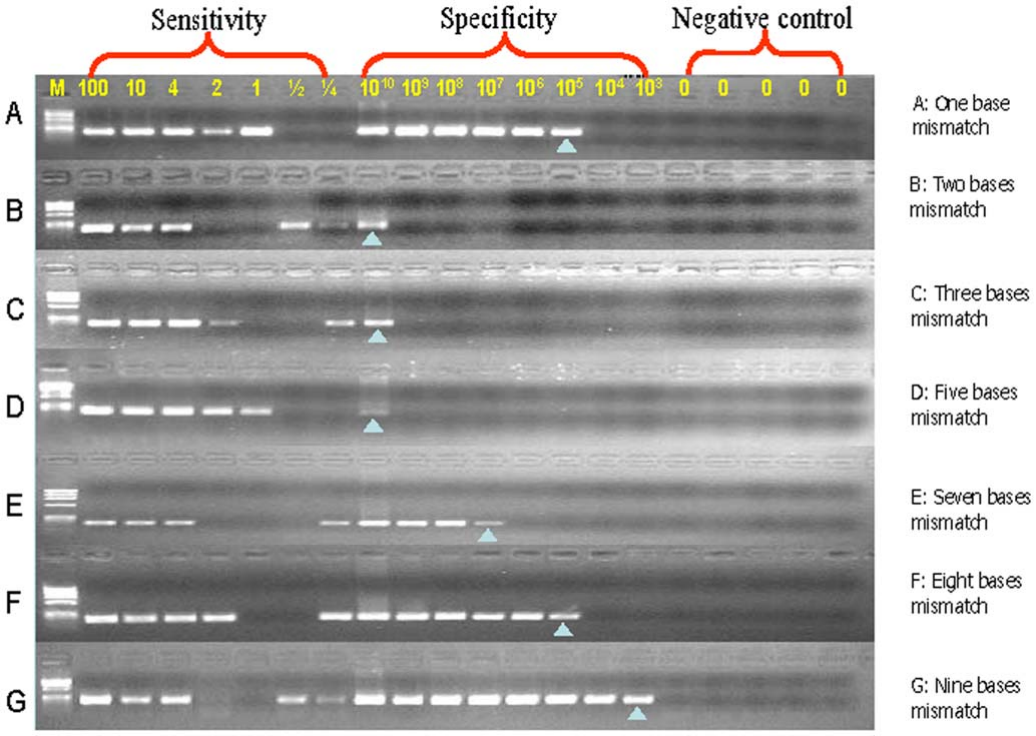
MAP analytical selectivity is related to the number of mismatched nucleotides using the *EGFR* 15 bp deletion as a model. The analytical selectivity of MAP is higher than 1×10^9^ when the number of mismatched nucleotides is 2–5, but sharply lower when the number of mismatched nucleotides is 7 or more. Analytical Sensitivity: Mutant DNA is serially diluted to 100, 10, 4, 2, 1, 1/2, 1/4 copies of template. The analytical sensitivity of the reaction is the minimum copy number of a mutant DNA that generates a detectable product when the primer matches the mutant template. The absence of a signal at one copy and the presence of a signal at ½ or ¼ copy are consistent with the Poisson distribution of expected signal resulting from dilution of DNA. Analytical Specificity: Wild type DNA is serially diluted from 10^10^ to 10^3^ copies. The analytical specificity of the reaction is the maximum copy number of the mismatched (wt) template that does not result in a detectable product when the primer mismatches the wild-type template. Analytical selectivity is the ratio of analytical specificity to analytical sensitivity. Negative controls do not contain targeted DNA. M: ФX174 DNA/HaeIII Marker.

The common 15 bp or 18 bp deletions in the epidermal growth factor receptor *(EGFR)* gene, commonly found in 5–20% of patients with non-small cell lung cancers [10], were chosen as models to explore the analytical sensitivity and analytical selectivity of MAP. In MAP, both downstream and upstream primers are blocked with a dideoxynucleotide (P*) and separated by 50 to 300 bp. The mutation-specific primers match the mutant and overlap the deletion junction so that two or more nucleotides mismatch the wild type sequence (Fig. 1A). When mutant-specific primers for the 15 bp deletion contain multiple mismatches (2–5 bases) with the wild-type template, the analytical selectivity of the assay is >10^9^ (Fig. 2B–D, Table S2) and 100,000-fold greater than that observed in PAP-A, which contains only one base mismatch at the 3′ end (analytical selectivity ≤10^4^) (Fig. 2A).

### MAP analytical selectivity is related to the number of mismatched nucleotides

The analytical sensitivity of detection in the MAP assay is one molecule for all assays (Fig. 2B–G). The Poisson nature of the serial dilution profiles (see **Methods**) supports the accuracy of copy number quantitation; e.g., sometimes 1 copy number does not amplify, whereas ½ or ¼ copy number does. Figure 2 provides an example of this. MAP can reproducibly amplify single molecules to ∼200 ng product in the presence of carrier genomic DNA or RNA by an estimated four trillion-fold without nesting. This yield is at least 100-fold better than routine PCR, presumably because the MAP primers are inert until activated and extended on their cognate template.

The analytical selectivity of one, two, three, five, seven, eight and nine mismatched nucleotides was tested (Fig. 2A–G, Table S2). Analytical selectivity is optimal with mismatches of 2 to 5 nucleotides (Fig. 2B–D). Analytical selectivity decreased dramatically with 7 or more mismatches. Since activation of P* can be inhibited even by single base mismatches up to 15 nucleotides from the 3′ end [15], it was hypothesized that the loss of analytical selectivity is due to the ability of trace amounts of unblocked oligonucleotides to artificially create the deletions by looping out the 15 nucleotides in wild-type DNA with increasing frequency as the heteroduplex of primer and loop-containing wild-type template is stabilized by longer strings of matching nucleotides beyond the deletion site (Fig. S2). Consistent with this hypothesis, i) hot-start MAP increased the analytical selectivity of the eight nucleotide mismatches from >10^4^ to >10^6^ (data not shown); and ii) sequence analyses indicate that the false positives with a PAP-A assay for a 15 bp deletion were due to one base misincorporation (Fig. S2 A) while the false positives with MAP were consistent with slipped heteroduplexes and mismatch pyrophosphorolysis (Fig. S2 B–D).

### MAP assay analytical selectivities generally are >10^9^ and analytical sensitivities are one molecule

**Figure 3:**
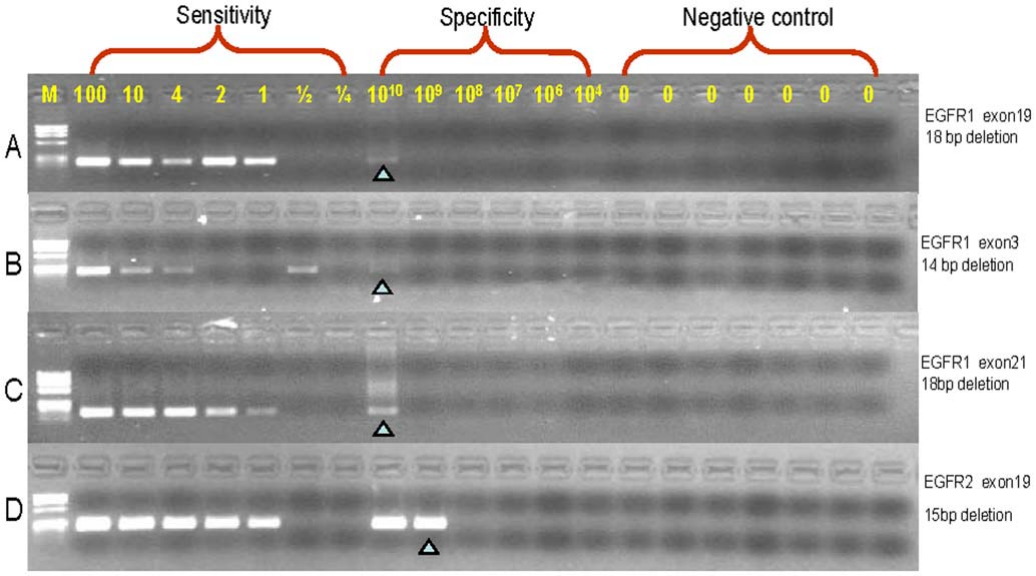
MAP detects deletions in human DNA with an analytical selectivity >10^8^. Four assays were established to test the MAP analytical sensitivity and analytical specificity (labeled by copy number). The analytical sensitivities of each assay are one copy and analytical specificities are >1×10^8^ copy. From top to bottom panels, primer mismatches with the wild type template were 4, 4, 4 and 6 nucleotides, respectively. The presence of a signal at a mean of one copy of the mutant template is predicted to be 63% based on the Poisson distribution due to random sampling, consistent with the absence of signal in some reactions with one copy of mutant template.

**Figure 4:**
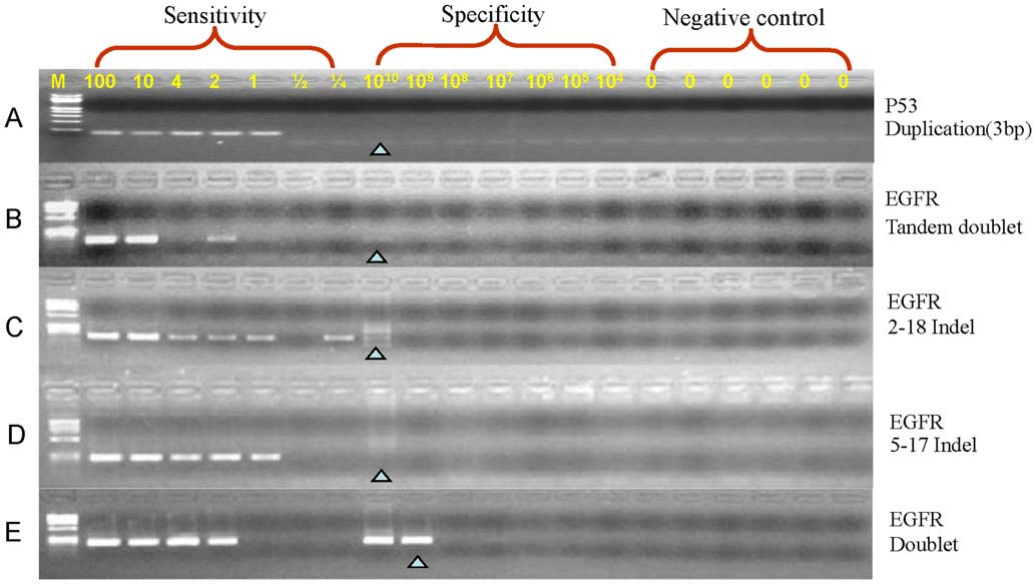
MAP detects insertions and complex mutations with an analytical selectivity >10^8^. The analytical selectivity of MAP for detecting an insertion in the *p53* gene (A) and complex mutations in the *EGFR* gene (B–E) is greater than 1×10^8^ in five different assays. In each assay, the primer contained 2–4 mismatches with the wild type sequence. The mutations detected were the following: *P53* gene: (A) Duplication: c.720_721insAGT; *EGFR* gene: (B) Tandem-base mutation: c.2154G>T, 2155G>T; (C) 2–18 Indel: c.2239–2258delinsCA; (D) 5–17 Indel: c.2237–2253delins TTGCT; (E) Doublet: c.2574T>G, c.2580A>T.

The MAP analysis of other deletions in the *EGFR*, *EGFR2* and *p53* genes in human or rat demonstrated analytical selectivities >10^8^–10^9^ when P* mismatched the wild type sequences by 3–5 nucleotides (Fig. 3, Table S3). Four assays with 4–5 mismatches had analytical selectivities >10^9^ and one assay with six mismatches had an analytical selectivity >10^8^. MAP was also demonstrated to detect four additional types of mutations with multiple base mismatches with respect to the wild type sequence. The analytical selectivity of MAP was >10^10^ for an insertion, >10^9^ for a tandem base mutation and for two indels, and >10^8^ for a doublet (two single base substitutions separated by 5 bases) (Fig. 4, Table S3).

A significant fraction of doublets, those closely clustered, can be assayed with high analytical selectivity by MAP [14]. For the *EGFR* doublet mutation at 2574 T>G and 2580 A>T, the analytical selectivity is 10^8^. This reflects the inhibition of P* activation by a mismatch six bases downstream from the 3′ end, consistent with previous data that mismatches far away from the 3′ end substantially inhibit the activation of P* [15]. Altogether, 15 MAP assays with 2–6 bp mismatches provided an analytical selectivity >10^8^–10^10^ and an analytical sensitivity of one molecule to detect MIDIs (Tables S2, S3).

### Proof of Principle: Direct analysis of in-vivo tissue mutagenesis – EGFR 15 and 18 bp deletions are not found in 8×10^7^ normal human lung cells

Until now, specialized genetic constructs such as Big Blue mice or selective medium that can identify mutations in limited cell types have been required for direct examination of *in vivo* mutagenesis in tissues. MAP assays can allow MIDIs to be analyzed directly in the species of interest. In an illustrative application, we tested as a hypothesis of interest that the *EGFR* 15/18 bp deletions found in 5–20% of non-small cell lung cancers might have arisen during the development of normal lung tissue and been enriched due to a selective replication advantage [10]. The common 15/18 bp *EGFR* deletions were not observed in a total of 8×10^7^ genomes from normal human adult lung (4 normal lung samples ×10^7^ copies of gDNA per sample ×2 assays for each lung) or in a total of 2×10^8^ genomes from the blood of normal individuals (10 blood samples ×10^7^ copies of gDNA per sample ×2 assays for each blood sample) (Fig. S3, Table S4). The analogous *EGFR2* gene 15 bp deletion in exon19 and an artificial 14 bp deletion in *EGFR* exon 3 were not detected in a total of 1.4×10^8^ genomes (10 blood samples ×10^7^ copies of gDNA per sample +4 lung samples ×10^7^ copies of gDNA per sample). Controls spiked with each of these two deletion mutations demonstrated that one mutant molecule can be detected in 1.7–3.3 µg gDNA (0.5–1×10^6^ genomes) and that the samples lacked inhibitors at the concentrations of genome utilized. No false positives were detected in multiple negative controls (no template DNA; mushroom DNA) showing that no contamination occurs during PCR set-up or DNA extraction.

### Proof of Principle: Direct analysis of in-vivo tissue mutagenesis – EGFR 15 and 18 bp deletions are not found in 5×10^7^ normal rat lung cells

Recently, *EGFR* mutations in the kinase domain (exons 18–21) were also found in 14% of lung adenomas/adenocarcinomas from *FEN1* mutant knock-in mice [20]. For the rat, MAP assays were developed for the *EGFR* 15 and 18 bp deletions homologous to those found in human. The *EGFR* 15 and 18 bp deletions in rat were not found in 10^7^ genomes from 5 normal rat lungs (5×10^7^ cells, in total) and livers, nor was a 15 bp deletion in rat *EGFR2* (homologous to the rat *EGFR* 15 bp deletion region) (Table S4).

### Proof of principle: High throughput population screening for germline and early embryonic cancer syndrome mutations

A family with a germline missense mutation in *EGFR* has a dramatic lung cancer phenotype [21]. An individual mosaic for an *EGFR* mutation may be at increased risk for lung cancer. If the *EGFR* deletions were hotspots of mutations by a novel mechanism, they may be predisposed to occur early in embryogenesis. Such individuals might be at an increased risk for lung cancer, but the mutation would not be detected by conventional screening methods.

Sixty-four hundred unrelated control DNA samples (86.8% European Caucasians, 4.3% Hispanics, 2.2% Asians, 2% Blacks, 2% Mestiza Columbian, 0.8% American-Indians, and 1.9% of unknown ethnicity) available in the laboratory were analyzed with MAP for the two common 15/18 bp deletions [22]. Samples were diluted to 200 ng/µl. These samples were pooled into groups of 400 individuals. 800 ng of genomic DNA were analyzed per pool (600 genomes per individual). No germline or mosaic mutation for any of the tested *EGFR* mutations was found in “gene pool” analysis with MAP (Fig. S4).

### Proof of Principle: Personalized monitoring of disease recurrence or therapy in the plasma and cellular components of blood – Cancer mutation signatures detected in six patients with non-metastatic breast cancer

**Figure 5:**
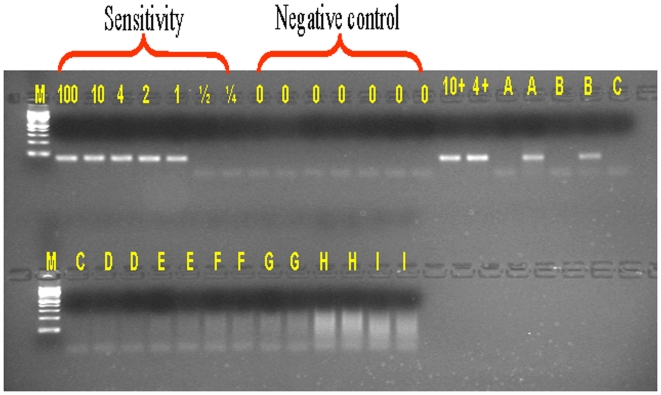
Cancer signature mutation was identified in 0.5 ml plasma at pretreatment and within 24 hours of initial chemotherapy in a patient with non-metastatic breast cancer. The MAP analytical sensitivity is demonstrated to be one copy. The positive controls (10+ and 4+) show no inhibitor when 10 or 4 copies of mutant templates are added to plasma DNA derived from A. Blood samples were obtained at the following times: A, B: at pretreatment and within 24 hours after cycle 1 chemotherapy. C, D: at pretreatment and within 24 hours after cycle 2 chemotherapy. E, F: at pretreatment and within 24 hours after cycle 3 chemotherapy. G, H: at midtreatment and within 3 weeks after surgery. Lane I is a control assay using crab gDNA extracted simultaneously with plasma to rule out contamination occurring during DNA extraction. For each time point, multiple duplicate reactions were performed (see Table 1). Only two reactions per time point are shown in this Figure.

MAP may be used for monitoring of therapy or recurrence of cancer in the cellular and plasma compartments of blood by using the mutation signature of the tumor. Analysis of tumor biopsy tissues from a woman with stage II breast cancer who underwent three cycles of neoadjuvant chemotherapy with doxorubicin, docetaxel, and cyclophosphamide (TAC) revealed a 3 bp somatic insertion in the *p53* gene (c.720_721insAGT, p.240dupS). A MAP assay was designed to determine whether the 3 bp insertion could be detected as a personalized marker for the tumor. The analytical sensitivity was one molecule and the analytical selectivity >1×10^10^ (Fig. 4A). The cellular compartment of blood was analyzed as an indicator of circulating tumor cells and the plasma was analyzed as an indicator of apoptotic/necrotic cancer-shedding membrane-encapsulated short DNA fragments in the circulation [23], [24].The mutation frequency was estimated at about 4 molecules per milliliter of plasma at pretreatment and within 24 hours after first treatment. After completion of neoadjuvant therapy, breast preserving surgery failed to demonstrate any pathological evidence of remaining tumor. The tumor-specific mutation became undetectable prior to the second cycle of neoadjuvant chemotherapy and remained undetectable at nine subsequent time-points spanning12 months of follow-up (Fig. 5, Table 1), consistent with the absence of clinical recurrence. The MAP assay was reproducible. Three independent DNA extractions and assays yielded the same results. The MAP assays were performed without knowledge of the clinical phenotype: Stage II disease, about 3.5 cm of tumor pre-therapy (Table 1), good response to neoadjuvant chemotherapy and continued remission at 13 months post diagnosis.

**Table 1:** Cancer mutation signature can be reproducibly detected in blood in patients with stage II or III breast cancer.

			Plasma			Cellular fraction		
Time-points^a^	Course	Days	Plasma Volume (ml)	Mutation signature per reaction^c^	Mutation frequency (per ml plasma)	Blood volume ml (molecules DNA)	Mutation signature per reaction	Mutation frequency (per ml blood)
Case-1^b^								
1	pre-treatment	0	3	7/11 (1.01)^d^	3.7	1 (1×10^7^)	0/20	0
2	cycle 1(post)	1	3	9/13 (1.18)^d^	5.1	1 (1×10^7^)	0/20	0
3	cycle 2 (pre)	18	3	0/13	0	1 (1×10^7^)	0/20	0
4	cycle 2 (post)	19	3	0/13	0			
5	cycle 3 (pre)	39	3	0/13	0			
6	cycle 3 (post)	40	3	0/13	0			
7	mid treatment	60	3	0/13	0			
8	3 weeks after surgery	128	3	0/13	0			
9	3 month F/U	218	1	0/2^e^	0			
10	6 month F/U	308	1	0/4^e^	0			
11	9 month F/U	398	1	0/2^e^	0			
12	12 month F/U	488	1	0/2^e^	0	1 (1×10^7^)	0/20	0
Case-2^b^								
1	pre-treatment	0	0.01	2/10(0.223)^d^	223	1 (1×10^7^)	2/20(0.105)^d^	2.1
2	3 weeks after surgery	138	0.01	2/10(0.223)	200	1 (1×10^7^)	5/20(0.288)	5.8
3	3 month F/U	228	0.01	1/10(0.105)	105	1 (1×10^7^)	2/20(0.105)	2.1

a: Blood samples 1–8 and 10 in case-1 and 1–3 in case-2 were frozen, thawed, and centrifuged. DNA was extracted from the cell pellet and from the supernatant, which contained plasma and lysed red cells. Blood cells for time points 9, 11, and 12 were centrifuged when fresh and the plasma was removed.

b: The patient (case-1 with c.720_721insAGT in p53 gene) remained disease free at the end of follow-up. The other patient (case-2 with c.165_166delTG in p53 gene) died of progressive disease.

c: The number of positive signals appearing in the total number of MAP reactions per time point.

d: The expected average number of mutants per reaction is estimated using a formula (the frequency of zero mutants per reaction = e^−x^, where x is the average number of mutants per reaction), assuming that: the mutant distributes in the reaction according to a Poisson distribution; if one or more mutants are in the reaction, the amplification is positive; and if zero mutants are in the reaction, it is negative.

e: Follow-up assays were performed with 1 ml plasma instead of 3 ml, as in the earlier assays. Fewer reactions were run, perhaps decreasing the analytical sensitivity of the measurement.

Another patient, who had stage III inflammatory breast cancer, was found to harbor a 2 bp deletion (c.165_166delTG) within the *p53* gene. The three blood samples available revealed the cancer signature in both the blood and cellular compartments (Table 1). Approximately 200 copies of the mutation signature were estimated per ml of plasma and persisted at 3 weeks after surgery and at the 3 month follow-up. Despite the large tumor load, there was very little mutation signature in the cellular compartment compared to the plasma compartment (Table 1). Indeed, some or all of the mutation signature in the cellular compartment may reflect contamination from plasma, i.e., about 1% plasma contamination in the buffy coat fraction could account for the cellular compartment values seen. Pathologic assessment of the patient's mastectomy specimen following neoadjuvant therapy revealed that virtually the entire breast mass consisted of malignant cells, indicating minimal response to neoadjuvant therapy. Shortly after surgery, metastases were found. The patient died of progressive disease.

In addition to these two patients, four others with stage II or III breast cancer had tumor-specific *p53* mutations identified and MAP assays developed for their analysis in blood (Table 2, Supplementary Fig.S5). In total, four of the six patients (ID # 1, 3, 4, 6) had detectable levels of tumor-specific mutation in plasma at diagnosis, but the levels fell to zero after therapy resulting in clinical remission. However, the cancer mutation signature was present, even after therapy, in all samples from two other patients (ID # 2 and 5), reflecting tumor progression.

**Table 2:** The correlation of the copy number of cancer mutation signature and tumor load at diagnosis.

Patient ID	Stage	Size by RECIST criteria	*p53* gene mutation	Copy number of mutation signature per ml plasma	Copy number of mutation signature in cellular compartment per ml blood
1 a	Stage II	R: 3.5 cm	c. 720_721insAGT	4 b	0
2 a	Stage III	R 12 cm mass and 4 cm lymph in inflammatory breast cancer	c. 165_166delTG	223	2
3	Stage III	5.5 cm L breast/multiple nodes	c. 642_643delTA	10 b	0
4	Stage III	L 2 cm and 2.8 cm lymph node and inflammatory breast cancer	c. 581T>G	9 b	3
5	Stage III	L 6.5 cm cancer and 2.8 cm lymph node in axilla with several others	c.216_217insC	600	NA
6	Stage III	L 6.6 cm cumulative (multifocal) with 1.8 cm lymph node and other nodes	c. 723delC	6 b	NA

a Additional information about patients 1 & 2 is described in Table 1 (Cases 1 & 2).

b The cancer mutation signature decreased to 0 per ml plasma during therapy resulting in clinical remission.

c Abbreviations: NA- not available; L- left breast; R- right breast.

## Discussion

MAP, a highly sensitive assay for ultra-rare mutations, enhances the detection of MIDIs by using multiple oligonucleotide mismatches, enabling detection generally at one part in a billion. False positives seem to arise by the extremely rare serial coupling of two events: slipped heteroduplex formation and mis-pyrophosphorolysis within a distorted heteroduplex. The practical outcome is robust and routine detection of ultra-rare MIDIs and complex mutations with an analytical selectivity generally >10^9^ and an analytical sensitivity that is generally one molecule. MAP was found to be methodologically robust when utilized i) to detect *EGFR* mutations in lung tissue or ii) to detect *p53* breast cancer signatures in plasma and the cellular compartments of blood or iii) to screen for mosaicism for common *EGFR* mutations in a large population.

Three methods now provide a complete ultra-rare mutation detection platform: classic PAP-A for any type of mutation, Bi-PAP-A for single base substitutions, and MAP for MIDIs. A summary of the three methods, including their analytical selectivities and the types of mutations for which they are most useful, is shown in Table S5.

### MAP Assay: Limitations

The MAP assay is by far the most sensitive assay described thus far for detecting MIDIs. In the analytical specificity assays, however, there are technical limitations in using human genomic DNA. For example, 10^9^ copies of human genomic DNA is equivalent to 3,300 µg DNA, which requires a large amount of tissue. Addition of this quantity of DNA is not feasible for a typical reaction mixture of 25–50 µl. To avoid this problem, we constructed a plasmid (4∼5 kb) containing a 300∼400 bp segment identical to the human genomic DNA region of interest; the copy number for the wild-type sequence can now be elevated to 10^11^ per µl along with 10^6^ copies of total wild type genomic DNA. At least 10^9^ copies of genomic DNA is a preferable reconstruction experiment, but it is not technically feasible. However we note that certain human sequences are present within these reconstruction experiments at 10^12^ copies without interference with PAP analytical selectivity.

### Hypothesis to explain the high frequency of the three common EGFR somatic mutations observed in lung cancer

The approximately four trillion cells in adult lung contain essentially every possible mutation; depending on the base pair, frequencies typically range from 10^−7^–10^−10^. Our hypothesis states that the three *EGFR* mutations commonly seen in lung cancer (L858R and the 15 bp and 18 bp deletions), which comprise >80% of all *EGFR* mutations seen in lung cancer, occur due to selection of cells with these mutations during lung development, resulting in greatly increased frequencies of these mutations compared to similar types of non-selected mutations in *EGFR* and other genes.

### MAP in lung DNA: EGFR mutants are not detected by MAP in normal human or rat lung cells

The *EGFR* (exon 19) 15 bp or 18 bp microdeletions are commonly found in 5–20% of patients with non-small cell lung cancers [10]. As lung cancer accounts for about 1/3 of all cancer deaths, these deletions are involved in about 1.5–6% of all lethal cancer events. Neither the mutation mechanism nor the mechanism of oncogenesis of these frequent mutations is understood.

The sequence context of these deletions does not suggest an endogenous hotspot [25]. These deletions could be a mutation signature from some as yet unknown mutagenic agent. Alternatively, these somatic deletions could be rare events that occur early in lung development and that are then enriched due to a selective advantage, as observed in other systems [10], [26], [27]. The above hypotheses would predict that these deletions are potential cancer driver mutations rather than passenger mutations, and that normal lung may contain these deletions at some low frequency. Neither of the *EGFR* microdeletion mutations was detected in a total of 8×10^7^ genomic DNA copies in four normal human lungs or in a total of 2×10^8^ genomes in blood samples from 10 healthy patients, suggesting that the hypotheses are incorrect, at least at the mutation frequencies tested. Homologous deletions in similar copy numbers of genomic DNA from five normal rat lungs were also not detected. Additionally, no germline or mosaic mutation for any of the tested *EGFR* mutations was found in “gene pool” analysis with MAP. The data indicate that these deletions are unlikely to occur during embryogenesis or lung development. These results do not support the hypothesis of mutational mosaicism, followed by enrichment by selection.

Note that the hypothesis could still be correct if the frequency of the deletions was 10^−10^ and the enrichment was 500-fold, such that the mutations would be below the detection threshold of the experiment. The analytical selectivity of MAP would allow these hypotheses to be tested further by analyzing larger amounts of sample. Most pediatric leukemias are initiated *in utero* [26], [28]; it still remains possible that certain adult tumors could be initiated *in utero*.

### MAP in blood from women with breast cancer: Personalized tumor-specific cancer signatures are detected in six of six patients with non-metastatic breast cancer and their levels reflect disease course

Among several reported studies in colorectal cancer, lung cancer, and ovarian cancer patients, a tumor-specific *p53* mutation was detected in an average of 40%, 75%, and 26%, respectively, of the corresponding plasma samples (Table 3, Table S6). In the present study, the detection of *p53* tumor-specific mutations in plasma DNA samples from 100% of patients (6/6) with non-metastatic breast cancer is significantly higher than those observed in colorectal cancer (p = 0.006) and ovarian cancer (p = 0.007). However, the plasma levels of the lung cancer tumor signature seem generally higher, as about 75% were detected by SSCP or sequencing.

**Table 3:** Summary of p53 mutations detected in plasma of cancer patients with non-metastatic disease a.

Study^b^	# patients with tumor mutation	# patients with plasma mutation	% mutations (plasma/ tumor)
**Colorectal cancer**			
Hibi '98	10	7 c	70% c
Mayall '98	3	2	67%
Wang '04	31	9 c	29% c
Bazan '06	19	7	37%
**Total CRC**	**63**	**25**	**40%**
**Lung cancer**			
Andriani '04	26	19	73%
Gonzalez '00	6	5	83%
**Total lung cancer**	**32**	**24**	**75%**
**Ovarian cancer**			
Otsuka '04	12	2	17%
Swisher '05	60	17 d	28% d
**Total ovarian cancer**	**72**	**19**	**26%**

a The literature was reviewed for English language publications that provided the following information: (1) tumor stage: only non-metastatic tumors, Stages I-III, were included (for colorectal cancer, modified Dukes Stages A–C); (2) the number of tumor samples with identified p53 mutations; (3) the number of corresponding plasma DNA samples that were positive for the same personalized p53 mutation detected in the tumor. The literature review included publications cited within Fleischhacker and Schmidt (2007).

b References:

Andriani F et al. (2004).*Int J Cancer* **108:** 91–96.

Bazan V et al. (2006).*Ann Oncol* **17 Suppl 7:** vii84–vii90.

Gonzalez R et al. (2000).*Ann Oncol* **11:** 1097–1104.

Hibi K et al. (1998).*Cancer Res* **58:** 1405–1407.

Mayall F et al.(1998). *J Clin Pathol* **51:** 611–613.

Otsuka J et al. (2004). *Int J Gynecol Cancer* **14:** 459–464.

Swisher EM et al. (2005). *Am J Obstet Gynecol* **193:** 662–667.

Wang Q et al. (2003). *Int J Cancer* **106:** 923–929.

c Mutations were detected in serum, rather than plasma.

d Mutations were detected in serum or plasma.

The levels of plasma mutation signature depend on the rate at which tumor necrosis and apoptosis occur, the rate at which protected DNA fragments are generated, and the rate at which these are cleared from the circulation. Patient- or tumor-specific differences are possible, complicating the relationship between tumor mass and the molecules of the cancer signature. For a given tumor in a given patient, the tumor signal in blood may be proportional to tumor burden, unless drug therapy confounds the risk of tumor apoptosis/necrosis. Future studies with careful quantitation of tumor mass are needed to clarify these issues.

To detect the cancer signature in 10^7^ genomes (3.3 µg genomic DNA per tube x 10 tubes) requires an analytical selectivity of 10^8^ or 10^9^ if the false positive rates are to be kept at 10% or 1%, respectively. The MAP assay is exquisitely selective for MIDIs and other complex mutations. In the study herein, the level of mutant *p53* molecules (within a background of wild type *p53* molecules) in four of the six plasma samples was very low (Table 2) and would not be detectable by most of the common methods of analysis. Within the limits of sample size and unidimensional estimates of tumor sizes, the signal corresponds very roughly to the ratio of tumor load to body weight, as might be expected if plasma shedding due to apoptosis/necrosis was roughly similar to normal cells. Among the six patients in this study, initial plasma cancer mutation signature levels per ml varied 150-fold. The variation is not well correlated with tumor size, e.g., 100-fold variation in plasma levels occurs in two tumors of 6.5 centimeters in one dimension. However, the standard measure of tumor size, which is used for the RECIST criteria [29], is one dimensional and may well not reflect tumor volume accurately. A tumor-specific mutation signature was detected in the initial plasma samples from all patients, but in the cellular compartment of blood in only two patients, one of whom subsequently developed metastatic disease (Patient 2). It remains to be determined whether detectable *cellular* cancer mutation signature is an indicator of particularly poor prognosis.

Caveats to the correlation of the tumor-specific signal in plasma or the cellular compartment of blood with the actual tumor burden in the patient are (i) the possible underestimation of the tumor burden if the somatic tumor mutation is present only in some regions of the tumor and (ii) overestimation of the tumor burden if occasional somatic mosaicism occurs in normal tissue. Analysis of three to four tumor-specific somatic mutations will be helpful to rule out signal deriving partially from non-neoplastic cells or from somatic mutations present in only a subset of the neoplasm. However, microdissection and sequencing of breast cancer samples suggest that *p53* mutations are generally, if not virtually always, clonal within breast cancer [30], [31].

### Personalized Monitoring for early recurrence in cancer

Personalized monitoring for tumor response in the neoadjuvant and advanced setting, as well as for detection of early recurrence, may help optimize treatment by assessing the tumor signature in the cellular and plasma components of blood over time. Such monitoring may alleviate both under-treatment and over-treatment, especially in the adjuvant setting. It may allow response to therapy to be assessed and semi-quantitated in a manner superior to that achievable by imaging. Bi-PAP-A and especially MAP provide the technology for routinely accessing ultra rare mutations in the cellular and plasma components of blood. While it is possible that confounders of the measurement may limit its clinical utility, the tools are now available to make the clinical measurements. Such monitoring could be of help for treating a variety of adult and pediatric tumors. For example, in childhood cancers such as Ewing's sarcoma and rhabdomyoscarcoma, aggressive therapy can cure about 70% of patients, while toxicity claims another 10% and recurrence the remaining 20%. Roughly 1/3 of patients are appropriately treated and the remaining patients are either over-treated or under-treated.

### MAP in population screening: Screening to identify individuals with germline or mosaic mutations predisposing to disease

Preventive medicine promises to reduce the cost of healthcare. Below, we suggest that a national investment in generating pools of DNA from millions of individuals could provide a national DNA resource for effective mass population screening. We conclude that MAP, PAP and Bi-PAP-A may utilize this resource to facilitate cost-effective preventive medicine, e.g., see below.

MAP and PAP are synergistic. A duplex MAP assay for the common 15 and 18 bp deletions was performed as proof of principle of the multiplexing potential of MAP assays (data not shown). PAP assays have previously been shown to multiplex with sufficient rigor for dosage analysis [35]. MAP and Bi-PAP-A have the potential to screen for a cocktail of mutations in large populations for which early embryonic mutations (mosaicism) as well as germline mutations could be detected. Since P* primers generally do not form primer dimers in solution, highly multiplexed PAP reactions for hundreds or even thousands of specific mutations may be possible in solution. Additionally, microarrays may facilitate multiplexed amplification. Bi-PAP-A and MAP assays can work synergistically within one cocktail to detect point mutations and deletions/insertions/indels with ultra high analytical selectivity.

We present proof of principle that MAP/PAP cocktails have the potential for highly efficient screening for mosaicism. Inheritance of germline mutations in more than 2,800 genes cause documented genetic disease (see OMIM database: http://www.ncbi.nlm.nih.gov/omim/). Milder forms of these diseases can occur in mosaic individuals who have experienced a relevant mutation very early in embryogenesis. As illustrated by our analysis of two common mutations in the *EGFR* gene, we screened 800 ng of genomic DNA per pool (600 genomes per individual) in a pool of 400 individuals. Since a single cell contains 6.6 pg DNA (2 genomes), a mosaic mutation in an individual at a frequency of 1 in 300 cells could be detected. By scaling up the volume by 10-fold, 4,000 individuals can reasonably be screened in one reaction to detect a mosaicism frequency of 1 in 300 cells. Only 250 such reactions would be required to screen 1 million individuals. Further scaling up to test 40,000 individuals in a single reaction could screen 10 million in 250 reactions.

A cocktail of Bi-PAP-A and MAP could be used to screen for known super hotspots of mutation, i.e. the super hotspots causing achondroplasia, Apert syndrome, and DMD [32], [33], [34]. Achondroplasia and Apert syndrome, which are dominant, severe, highly penetrant diseases, are caused by only one or a few mutations for the overwhelming majority of patients. Individuals who are mosaic for these mutations may be mildly affected and at high risk for having offspring with the severe disease. If individuals with the above germline or somatic mutations were detected at a frequency of even 1 in 200,000, five such individuals could be detected per million individuals screened for an incremental cost of developing and performing the PAP assays of just a few thousand dollars.

### MAP for screening plasma in individuals for early detection of cancers

Personalized detection of early onset cancer and/or early cancer recurrence is an area of active investigation. A panel of 21 *p53* gene mutation assays (MAP or Bi-PAP-A) may detect the mutational signature in ∼30% of breast cancers, based on an examination of data from the IARC TP53 database (www-p53.iarc.fr). Analysis of 10 ml of plasma may be expected to detect one gram of solid tumors and possibly smaller tumor burdens. This assay might involve one multiplexed amplification [35].

For lung and pancreatic cancer, which account for about 35% of cancer deaths, PAP-based screening may be helpful, as no well accepted and cost effective screen for early tumors is available. The screening test would be developed to detect common somatic mutations found in a large percentage of these cancers. Normally these tumors are discovered at late stages and associated with poor prognosis. If the levels of mutation signature found in our sample of six breast cancers are typical, analysis of 10-fold more plasma (20 ml of blood) should detect the presence of tumors less than 1 gram. If the mutation signature increases in subsequent measurements, a search for the cancer may be cost effective in high-risk populations. MAP analysis of breast cancer suggests that roughly one gram of tumor is associated with about one molecule of tumor mutation signature in the plasma compartment of blood. However, the rate of false positives, which can result from occasional non-tumor mosaicism for one of the mutations in the cocktail, would need to be determined in an epidemiological trial.

The demonstration that *EGFR* deletions are not detected in normal lung and blood, coupled with the demonstration that remnants of presumptive apoptotic, necrotic cancer cells can be detected in early stage cancer, lead to the possibility that the common *EGFR* mutations could be used to detect the presence of early lung cancer in high-risk populations. A cocktail of MAP assays for the common 15/18 bp deletions, together with a Bi-PAP-A assay for the common L858R mutation, could detect about 70–80% of *EGFR* mutations constituting about 10% of total lung cancers [10], [36]. It remains for future epidemiological studies to determine if a MAP-based screen is cost effective for detecting early stage lung cancers when >90% could be surgically cured [37], [38], [39]. By identifying the *EGFR* 15/18 bp deletions in blood, the ultra high analytical selectivity of MAP could potentially be applied to early lung cancer detection or possibly facilitate more rational chemotherapy delivery by monitoring the efficacy of therapy or predicting recurrence.

### Conclusion

The analytical selectivity of MAP (generally one per billion) and analytical sensitivity of MAP (generally one molecule with a mutation) is demonstrated. Proof of principle is presented for three types of clinical applications. MAP was found to be methodologically robust when utilized i) to detect *EGFR* mutations in lung tissue; ii) to detect *p53* breast cancer signatures in plasma and the cellular compartments of blood; or iii) to screen for mosaicism for common *EGFR* mutations in a large population.

#### Note

While this manuscript was in process, Diehl et al [40] reported that the cancer mutation signature over the course of disease in colorectal cancer patients (16 stage IV, 1 stage II, 1 stage III) can be quantitatively detected. Although the method that was used (BEAMing) [3] may not be as selective as MAP, the study confirms the feasibility of personalized monitoring of cancer therapy and recurrence.

## Supporting Information

Table S1 (0.03 MB XLS)

Table S2 (0.02 MB XLS)

Table S3 (0.02 MB XLS)

Table S4 (0.02 MB XLS)

Table S5 (0.03 MB XLS)

Table S6 (0.02 MB XLS)

Figure S1 Schematic of the potential analytical specificities of PAP-A and Bi-PAP-A A: PAP-A: When a P* oligonucleotide is annealed to its complementary template, the 3′ terminal blocker can be removed by pyrophosphorolysis in the presence of pyrophosphate. The activated oligonucleotide can be extended by DNA polymerization (Left panel, specific amplification). Non-specific amplification (Non-specific, right panel, type I error) may occur at a frequency of 10^−5^, but it is not an efficient template for subsequent cycles. Significant non-specific amplification (Non-specific, right panel, type II error) requires mismatch pyrophosphorolysis followed by misincorporation by the DNA polymerase, an event with a frequency estimated to be 3.3×10^−11^. B: Bi-PAP-A, point mutation: Panel B shows Bi-PAP-A detection of a point mutation (T>A). The two P* overlap at their 3′ termini by one nucleotide to eliminate polymerase misincorporation (T>A; error rate: ∼10^−5^) at the mutation position during the opposite primer extension (the bypass reaction). C: Bi-PAP-A, deletion: When Bi-PAP-A strategy was applied to detect the *EGFR* 15 bp deletion, the downstream and upstream mutant-specific blocked primers are complementary at three nucleotides at the 3′ end of primers and may form primer dimers (acting as mutant templates) resulting in false positives due to >10^12^ molecules of the primers (2.5 µM) within the reaction.
(0.05 MB DOC)

Figure S2 Sequence analyses of false positive products show the two mechanisms limiting the PAP-A and MAP analytical specificity. A: False positive is a wild type sequence with one misincorporation (C>A) during the downstream primer extension; Sequence analysis shows a segment of size and sequence expected from wild type DNA with the predicted one misincorporation at the 3′ end of the deleted region (Arrow). B: False positive from two base mismatch primers is due to slippage of 31 bp upstream; C–D: 8 or 9 bases at the 3′ end of P* match with wild-type template with a loop out of 15 bp segment resulting in a false positive.
(0.03 MB PPT)

Figure S3 No detection of the EGFR 15 bp deletion in human lung by MAP in 4 normal lung samples and mushroom control. The common *EGFR* 15 bp deletion (sample ID1-4) was not found in normal lung from 1×10^7^ copies (0.5×10^6^ copies/tube ×20) of human lung tissues. The first two lanes in every sample and mushroom DNA are positive controls spiked with 10 and 4 copies of mutant templates, respectively. A∼T lanes indicate 20 parallel DNA reactions from the same sample containing 0.5×10^6^ copies genomes per tube. The first row shows analytical sensitivity assays and negative control assays performed simultaneously. The last row shows mushroom DNA control to monitor the contamination during DNA extraction.
(0.26 MB PPT)

Figure S4 No detection of mosaicism in a “gene pool” analysis of the EGFR 15/18 bp deletions in 6,400 individuals. The possibility of somatic mosaicism in 6400 control individuals was tested in leukocyte DNA. Sixteen pools, each containing DNA from 400 individuals at an aggregated concentration of 200 ng/µl, were analyzed by MAP for the *EGFR* 15/18 bp deletions. A series of analytical sensitivity controls and negative controls are shown for each deletion mutation. The first two lanes following the DNA size marker M (ΦX174 DNA/HaeIII) contain positive controls spiked with 4 and 2 copies of mutant templates, respectively. Lanes A–P contain the 16 pooled samples, each with DNA from 400 individuals. Somatic mosaicism for the *EGFR* 15 bp or 18 bp deletions was not detected in any sample.
(0.32 MB PPT)

Figure S5 Real-Time MAP shows a linear relationship in case-5 (also see Table 2). DNA from Patient #5 (see Table 2) with a *p53* gene mutation (c.216_217insC) was analyzed by real-time PCR on the BioRad RQ5 instrument. Real-time MAP shows a linear relationship between MAP cycle number and the log of the starting quantity (from 1 to ∼10,000 copies) (R^2^ = 0.992).
(0.12 MB PPT)
