# Supplementary material for: Analysis of Cancer Mutation Signatures in Blood by a Novel Ultra-Sensitive Assay: Monitoring of Therapy or Recurrence in Non-Metastatic Breast Cancer
Source: PLoS One. 2009 Sep 28;4(9):e7220. doi: 10.1371/journal.pone.0007220 (PMC2749210; doi:10.1371/journal.pone.0007220)
Supplement: Figure S1 — Schematic of the potential analytical specificities of PAP-A and Bi-PAP-A A: PAP-A: When a P* oligonucleotide is annealed to its complementary template, the 3′ terminal blocker can be removed by pyrophosphorolysis in the presence of pyrophosphate. The activated oligonucleotide can be extended by DNA polymerization (Left panel, specific amplification). Non-specific amplification (Non-specific, right panel, type I error) may occur at a frequency of 10−5, but it is not an efficient template for subsequent cycles. Significant non-specific amplification (Non-specific, right panel, type II error) requires mismatch pyrophosphorolysis followed by misincorporation by the DNA polymerase, an event with a frequency estimated to be 3.3×10^−11^. B: Bi-PAP-A, point mutation: Panel B shows Bi-PAP-A detection of a point mutation (T>A). The two P* overlap at their 3′ termini by one nucleotide to eliminate polymerase misincorporation (T>A; error rate: ∼10−5) at the mutation position during the opposite primer extension (the bypass reaction). C: Bi-PAP-A, deletion: When Bi-PAP-A strategy was applied to detect the EGFR 15 bp deletion, the downstream and upstream mutant-specific blocked primers are complementary at three nucleotides at the 3′ end of primers and may form primer dimers (acting as mutant templates) resulting in false positives due to >1012 molecules of the primers (2.5 µM) within the reaction. (0.05 MB DOC) [file pone.0007220.s007.doc]

*tccctcaccttcgg****………***

***atcaa*[ggaattaagagaagc]*aacatctccgaaagccaac***

**Mutation-specific primer**

5’

3’

**Deleted bases**

**A***

**Mutant-specific primer**

***T**

**T**

**T**

**G**

**A**

**A**

**C**

**C**
