# Supplementary material for: Analysis of Cancer Mutation Signatures in Blood by a Novel Ultra-Sensitive Assay: Monitoring of Therapy or Recurrence in Non-Metastatic Breast Cancer
Source: PLoS One. 2009 Sep 28;4(9):e7220. doi: 10.1371/journal.pone.0007220 (PMC2749210; doi:10.1371/journal.pone.0007220)
Supplement: Figure S2 — Sequence analyses of false positive products show the two mechanisms limiting the PAP-A and MAP analytical specificity. A: False positive is a wild type sequence with one misincorporation (C>A) during the downstream primer extension; Sequence analysis shows a segment of size and sequence expected from wild type DNA with the predicted one misincorporation at the 3′ end of the deleted region (Arrow). B: False positive from two base mismatch primers is due to slippage of 31 bp upstream; C–D: 8 or 9 bases at the 3′ end of P* match with wild-type template with a loop out of 15 bp segment resulting in a false positive. (0.03 MB PPT) [file pone.0007220.s008.ppt]

## Slide 1
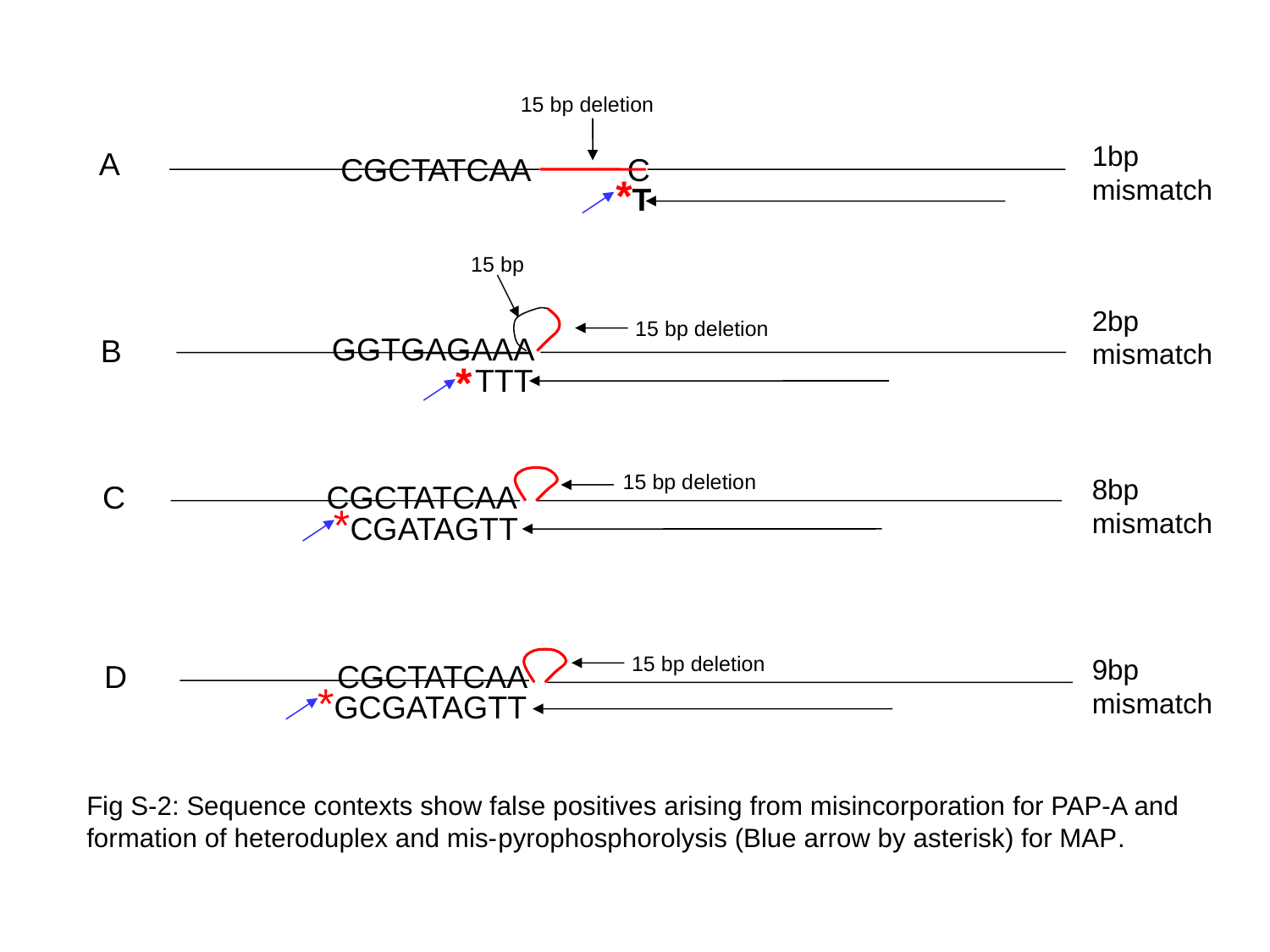

15 bp deletion
1bp mismatch
A
CGCTATCAA C
*T
15 bp
2bp mismatch
15 bp deletion
GGTGAGAAA
B
*
TTT
15 bp deletion
8bp mismatch
C
CGCTATCAA
*CGATAGTT
15 bp deletion
9bp mismatch
D
CGCTATCAA
*GCGATAGTT
Fig S-2: Sequence contexts show false positives arising from misincorporation for PAP-A and formation of heteroduplex and mis-pyrophosphorolysis (Blue arrow by asterisk) for MAP.
