# Supplementary material for: Analysis of Cancer Mutation Signatures in Blood by a Novel Ultra-Sensitive Assay: Monitoring of Therapy or Recurrence in Non-Metastatic Breast Cancer
Source: PLoS One. 2009 Sep 28;4(9):e7220. doi: 10.1371/journal.pone.0007220 (PMC2749210; doi:10.1371/journal.pone.0007220)
Supplement: Figure S3 — No detection of the EGFR 15 bp deletion in human lung by MAP in 4 normal lung samples and mushroom control. The common EGFR 15 bp deletion (sample ID1-4) was not found in normal lung from 1×10^7^ copies (0.5×10^6^ copies/tube ×20) of human lung tissues. The first two lanes in every sample and mushroom DNA are positive controls spiked with 10 and 4 copies of mutant templates, respectively. A∼T lanes indicate 20 parallel DNA reactions from the same sample containing 0.5×10^6^ copies genomes per tube. The first row shows analytical sensitivity assays and negative control assays performed simultaneously. The last row shows mushroom DNA control to monitor the contamination during DNA extraction. (0.26 MB PPT) [file pone.0007220.s009.ppt]

## Slide 1
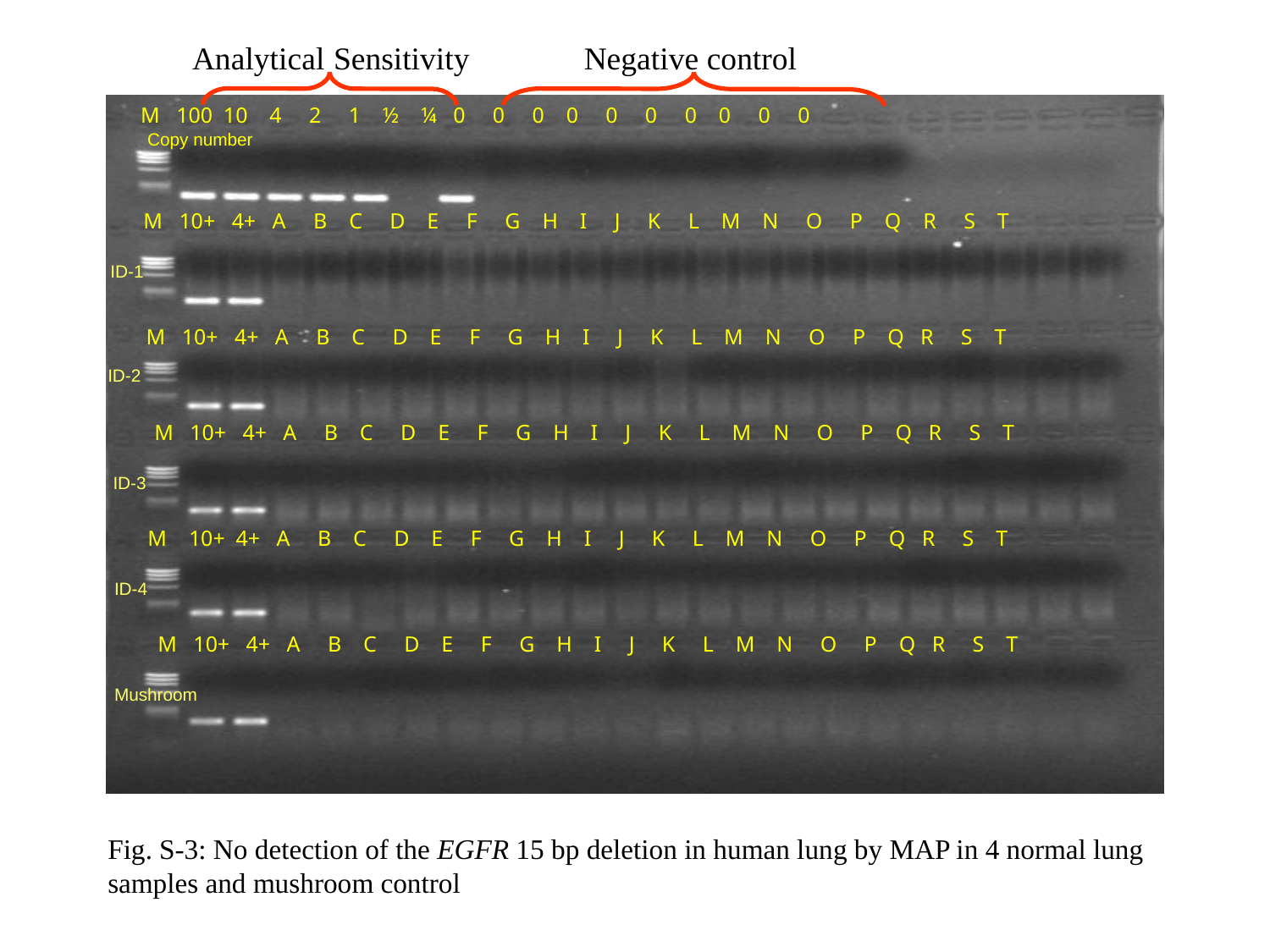

Analytical Sensitivity
Negative control
 M 100 10 4 2 1 ½ ¼ 0 0 0 0 0 0 0 0 0 0
Copy number
 M 10+ 4+ A B C D E F G H I J K L M N O P Q R S T
ID-1
 M 10+ 4+ A B C D E F G H I J K L M N O P Q R S T
ID-2
 M 10+ 4+ A B C D E F G H I J K L M N O P Q R S T
ID-3
 M 10+ 4+ A B C D E F G H I J K L M N O P Q R S T
ID-4
M 10+ 4+ A B C D E F G H I J K L M N O P Q R S T
Mushroom
Fig. S-3: No detection of the EGFR 15 bp deletion in human lung by MAP in 4 normal lung samples and mushroom control
