# Supplementary material for: Analysis of Cancer Mutation Signatures in Blood by a Novel Ultra-Sensitive Assay: Monitoring of Therapy or Recurrence in Non-Metastatic Breast Cancer
Source: PLoS One. 2009 Sep 28;4(9):e7220. doi: 10.1371/journal.pone.0007220 (PMC2749210; doi:10.1371/journal.pone.0007220)
Supplement: Figure S4 — No detection of mosaicism in a “gene pool” analysis of the EGFR 15/18 bp deletions in 6,400 individuals. The possibility of somatic mosaicism in 6400 control individuals was tested in leukocyte DNA. Sixteen pools, each containing DNA from 400 individuals at an aggregated concentration of 200 ng/µl, were analyzed by MAP for the EGFR 15/18 bp deletions. A series of analytical sensitivity controls and negative controls are shown for each deletion mutation. The first two lanes following the DNA size marker M (ΦX174 DNA/HaeIII) contain positive controls spiked with 4 and 2 copies of mutant templates, respectively. Lanes A–P contain the 16 pooled samples, each with DNA from 400 individuals. Somatic mosaicism for the EGFR 15 bp or 18 bp deletions was not detected in any sample. (0.32 MB PPT) [file pone.0007220.s010.ppt]

## Slide 1
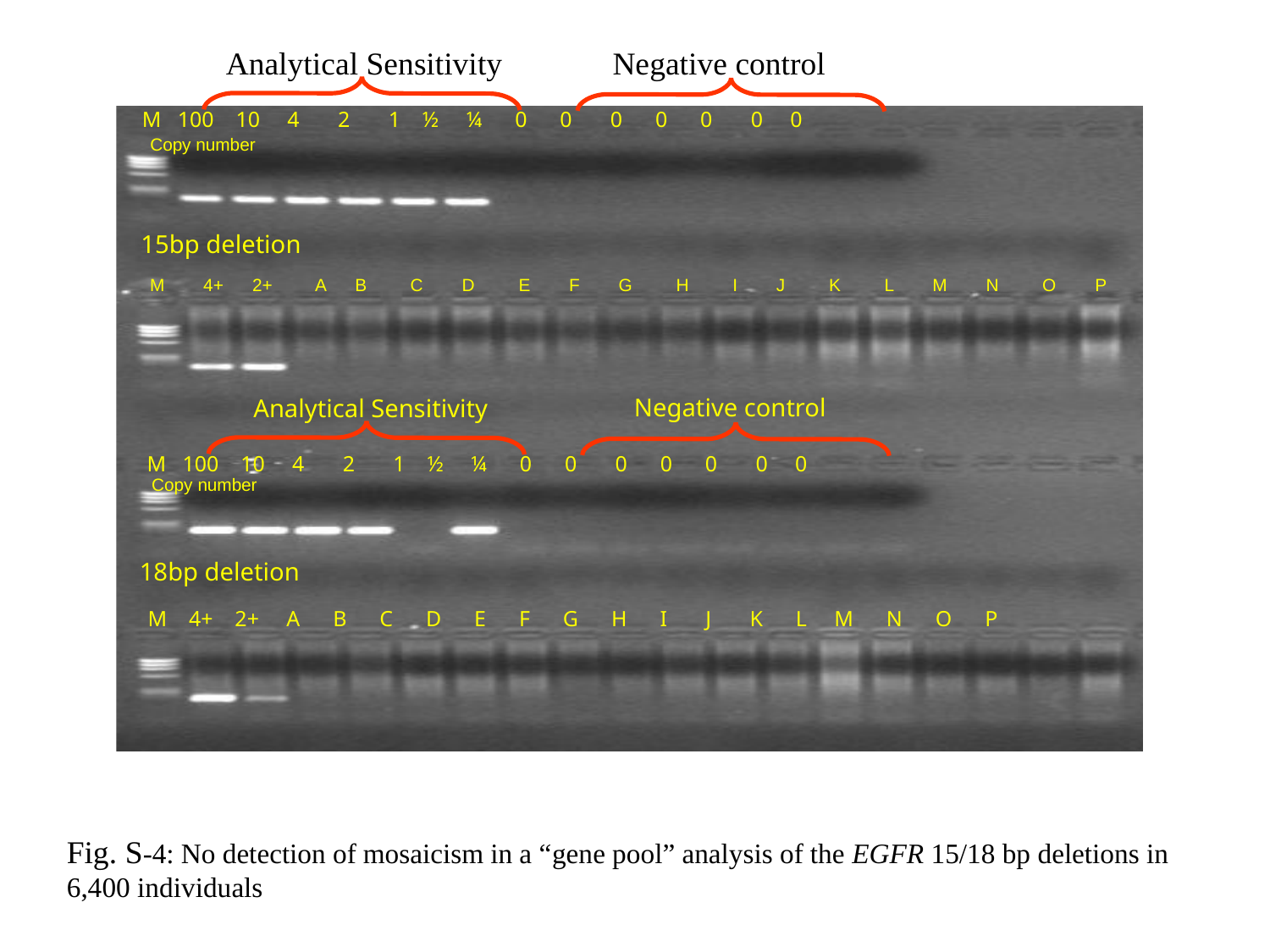

Analytical Sensitivity
Negative control
 M 100 10 4 2 1 ½ ¼ 0 0 0 0 0 0 0
Copy number
15bp deletion
 M 4+ 2+ A B C D E F G H I J K L M N O P
Negative control
Analytical Sensitivity
 M 100 10 4 2 1 ½ ¼ 0 0 0 0 0 0 0
Copy number
18bp deletion
 M 4+ 2+ A B C D E F G H I J K L M N O P
Fig. S-4: No detection of mosaicism in a “gene pool” analysis of the EGFR 15/18 bp deletions in 6,400 individuals
