# Supplementary figures and images for: Analysis of Cancer Mutation Signatures in Blood by a Novel Ultra-Sensitive Assay: Monitoring of Therapy or Recurrence in Non-Metastatic Breast Cancer
Source: PLoS One. 2009 Sep 28;4(9):e7220. doi: 10.1371/journal.pone.0007220 (PMC2749210; doi:10.1371/journal.pone.0007220)

## Slide 1
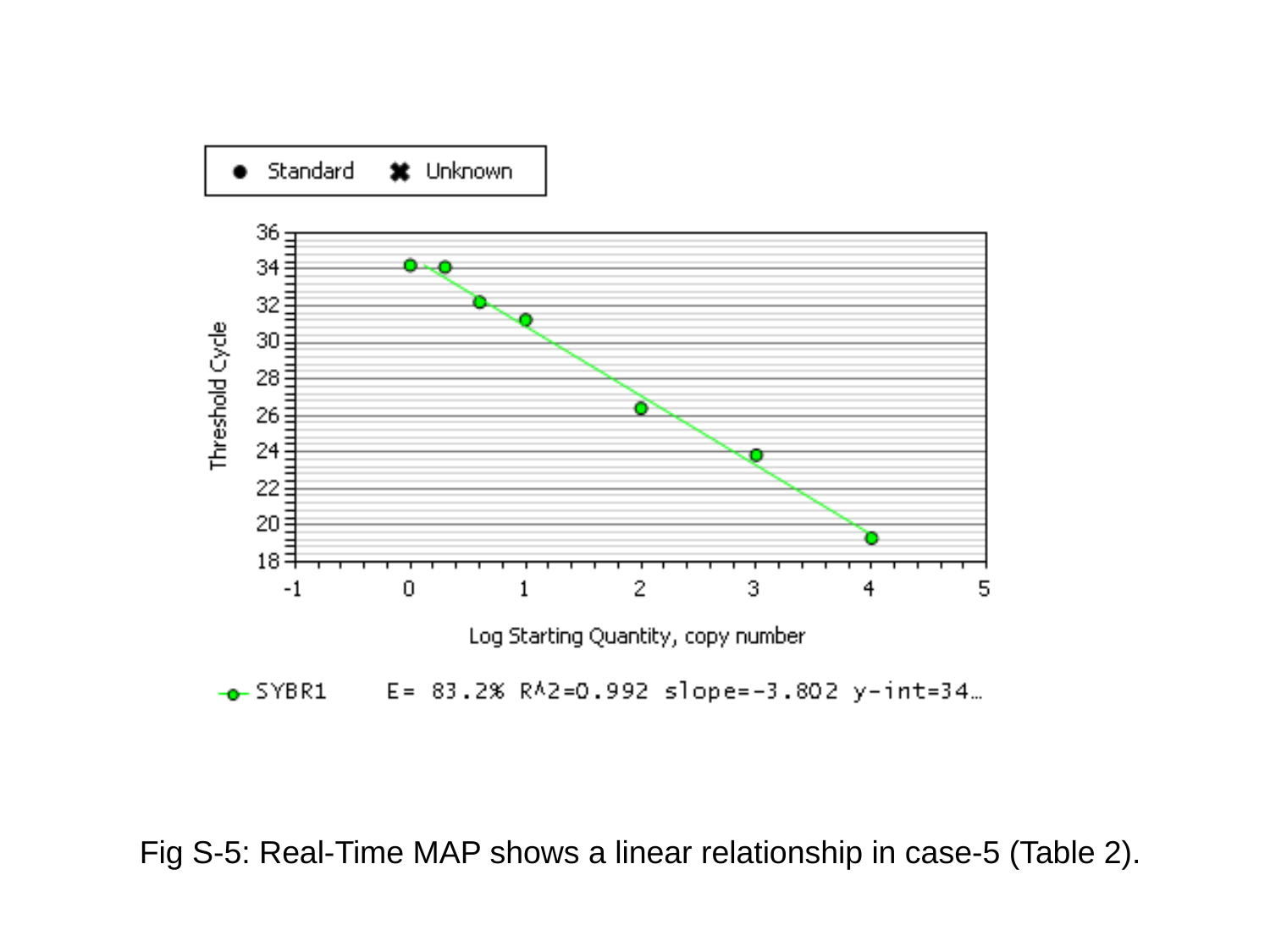

Fig S-5: Real-Time MAP shows a linear relationship in case-5 (Table 2).

Supplement: Figure S5 — Real-Time MAP shows a linear relationship in case-5 (also see Table 2). DNA from Patient #5 (see Table 2) with a p53 gene mutation (c.216_217insC) was analyzed by real-time PCR on the BioRad RQ5 instrument. Real-time MAP shows a linear relationship between MAP cycle number and the log of the starting quantity (from 1 to ∼10,000 copies) (R2 = 0.992). (0.12 MB PPT) [file pone.0007220.s011.ppt]
